# Development and validation of an interpretable machine-learning model for predicting treatment failure in severe trauma patients

**DOI:** 10.3389/fmed.2026.1919742

**Published:** 2026-09-07

**Authors:** Qiang Shi, Yun Liu, Jiahui Shen, Fei Yin, Zhenguo Qiao, Xie Shen, Jun Zhou, Deli Xu, Teng Zhang, Chengzhi Xu

**Affiliations:** 1 Department of Emergency, Suzhou Ninth People’s Hospital, Suzhou Ninth Hospital Affiliated to Soochow University, Suzhou, China; 2 Department of Emergency Intensive Care Unit, Suzhou Ninth People’s Hospital, Suzhou Ninth Hospital Affiliated to Soochow University, Suzhou, China; 3 Department of Gastroenterology, Suzhou Ninth People’s Hospital, Suzhou Ninth Hospital Affiliated to Soochow University, Suzhou, China

**Keywords:** machine learning, predictive modeling, prognosis, trauma, treatment failure

## Abstract

**Objective:**

This study aims to develop and validate machine learning (ML) models for predicting treatment failure in trauma patients using comprehensive clinical and laboratory variables, and to identify key prognostic features.

**Methods:**

A retrospective cohort of 318 trauma patients was included. We included 44 characteristics, and the primary outcome was treatment failure at hospital discharge, defined as in-hospital death, an unimproved or worsened discharge status, discharge against medical advice or withdrawal of active treatment because of critical illness, or a GOS score of 1–3 in patients with concomitant traumatic brain injury. The dataset was randomly divided into a training set (70%) and a test set (30%). Features were selected via least absolute shrinkage and selection operator (LASSO) regression in the training set. 5 ML models—Decision Tree (DT), Random Forest (RF), Support Vector Machine (SVM), k-Nearest Neighbors (KNN), and Extreme Gradient Boosting (XGBoost)—were trained and evaluated. The optimal model was interpreted using SHapley Additive exPlanations (SHAP) analysis.

**Results:**

LASSO regression selected 13 candidate predictors for model development in the training set. The RF model demonstrated the best performance in the test set, with an AUC of 0.967 (95% CI: 0.936–0.997), sensitivity of 1.000, specificity of 0.855, and F1-score of 0.776. SHAP analysis identified Glasgow Coma Scale (GCS) score as the most influential predictor, followed by Multiple Organ Dysfunction Syndrome (MODS), Acute PHysiology and Chronic Health Evaluation (APACHE II) score, Injury Severity Score (ISS), and creatine kinase (CK). Higher APACHE II and ISS scores were positively associated with treatment failure, while higher GCS, absence of MODS and CK levels correlated with reduced risk.

**Conclusion:**

Utilizing multiple trauma severity scores, laboratory parameters, and machine learning algorithms, we developed a predictive model to identify trauma patients at risk of treatment failure within the first 24 h of admission. Among the algorithms evaluated, RF model demonstrated superior internal discriminative performance in our single-center cohort. SHAP interpretability analysis reveals the core prognostic value of GCS, MODS, APACHE II, ISS, and CK, providing a potential transparent auxiliary tool for clinical risk stratification. However, its generalization performance still needs multi center external validation.

## 1 Introduction

Trauma presents a profound challenge to global health systems, contributing heavily to both fatal outcomes and persistent impairment. Data from the 2021 Global Burden of Disease Study reveals that from 2010 to 2021, the global age-standardized Disability-Adjusted Life Year (DALY) rate attributed to injuries witnessed a significant decline of 24.0%, with the estimated reduction ranging between 20.7% and 27.2% (1). Given the complex and evolving pathopHysiology of trauma, delivering precise prognostic assessments at an early stage represents a vital—though particularly difficult—aspect of patient care (2). The ability to forecast therapeutic outcomes in a timely manner is essential: it allows for more efficient use of critical care resources, informs tailored treatment approaches, and thereby enhances prospects for survival and meaningful functional restoration.

Traditionally, the prediction of clinical outcomes in trauma has been largely dependent on established statistical methodologies, including logistic regression and integrated scoring frameworks like the Injury Severity Score (ISS) and the Acute PHysiology and Chronic Health Evaluation (APACHE) system (3). However, relying solely on these variables cannot accurately predict clinical outcomes in trauma patients. In fact, multiple clinical biochemical indicators are closely correlated with patient prognosis (4). It is worth noting that traditional statistical analysis may not fully capture the complex nonlinear interactions between the large demograpHic, pHysiological, and laboratory variables of trauma patients. Machine learning (ML), an advanced branch of artificial intelligence, has fundamentally transformed clinical prediction modeling. By leveraging its capacity to process and interpret complex, multi-dimensional datasets, ML can detect subtle and intricate patterns that remain beyond the reach of traditional statistical methodologies (5). In recent years, ML technology has been increasingly used to predict the prognosis of trauma patients (6–8).

Therefore, this study aims to develop a series of ML models using a comprehensive set of clinical variables and select the one with the best performance to predict binary outcomes of successful treatment for trauma patients and identify the most influential predictive features (9).

## 2 Methods

### 2.1 Study population

This retrospective study screened adult patients with severe trauma admitted to the ICU/EICU. Adult patients with severe multiple trauma were eligible if they met the following criteria: (1) injuries resulting from a single traumatic event involving two or more anatomical regions; and (2) an Injury Severity Score (ISS) ≥ 16. Exclusion criteria encompassed: (1) age <18 years; (2) discharge or death before completion of relevant clinical examinations; (3) pre-existing comorbidities that could substantially confound the study outcomes, including severe cardiovascular disease, hepatic or renal failure; (4) coagulation disorders such as leukemia or hemopHilia; (5) incomplete or missing imaging data; (6) prehospital time exceeding 24 h.

A total of 331 patients were identified through preliminary screening from ICU and EICU at Suzhou Ninth People's Hospital. Following the application of the above criteria, 10 patients were excluded for the following reasons: ISS <16 (*n* = 4), combined severe cirrhosis and hepatic failure (*n* = 1), combined severe coronary heart disease and heart failure (*n* = 1), missing imaging data (*n* = 3), and prehospital time > 24 h (*n* = 1). Additionally, 3 patients were further excluded due to incomplete clinical variables. Consequently, 318 patients were ultimately included in the final analysis cohort.

GCS was defined as the earliest reliable score obtained before administration of sedative or neuromuscular blocking agents and before endotracheal intubation. For patients who underwent prehospital intubation, the last documented pre-intubation GCS score from the prehospital medical record was used. Shock was defined as a clinician-documented diagnosis of shock present at admission, based on the admission medical record and the initial assessment by the treating trauma team.

This study was reviewed and approved by the Ethics Committee of Suzhou Ninth People's Hospital (Approval Number: KYLW2026-022-01). Given that this is a retrospective study and the data has been de-identified, making personal identification impossible, informed consent has been waived. The privacy rights of all human subjects have been strictly observed, and all data has been handled in an anonymized manner to ensure that no personal information is disclosed.

### 2.2 Outcome

Patients were categorized according to their clinical status at hospital discharge. The primary endpoint was treatment failure at discharge. For patients without traumatic brain injury, treatment success was defined as survival to hospital discharge with the discharge status documented as “cured” or “improved,” whereas treatment failure was defined as in-hospital death, a discharge status documented as “unimproved” or “worsened,” or discharge against medical advice/withdrawal of active treatment because of critical illness. For patients with concomitant traumatic brain injury, neurological outcome at discharge was classified using the Glasgow Outcome Scale (GOS), with GOS scores of 4–5 classified as treatment success and GOS scores of 1–3 classified as treatment failure. Treatment failure was encoded as 1 and treatment success as 0.

### 2.3 Feature

A comprehensive set of clinical variables, including demograpHic characteristics, disease status, severity scores, vital signs, and laboratory parameters, were collected for each patient upon admission. The variables were selected based on clinical relevance and availability in trauma care settings. DemograpHic characteristics comprised gender and age. Lifestyle factors included smoking and drinking status. Disease status was assessed by the presence of multiple organ dysfunction syndrome (MODS) and shock. Disease severity was evaluated using three established scoring systems: the APACHE II score, the Glasgow Coma Scale (GCS) score, and the ISS. Vital signs recorded were pulse (P, beats per min), respiratory rate (R, breaths per min), mean arterial pressure (MAP, mmHg), and body temperature (T, °C). Laboratory parameters encompassed a wide spectrum of tests reflecting pHysiological and metabolic status: arterial pH, potassium (K, mmol/L), lactate (LAC, mmol/L), red blood cell count (RBC, ×10^12^/L), hemoglobin (HB, g/L), hematocrit (HCT, %), platelet count (PLT, ×10^9^/L), albumin (ALB, g/L), cardiac troponin I (cTnI, μg/L), myoglobin (MYO, μg/L), thrombin time (TT, s), prothrombin time (PT, s), activated partial thromboplastin time (APTT, s), fibrinogen (Fib, g/L), D-dimer (DD, mg/L FEU), creatine kinase (CK, U/L), creatine kinase-MB isoenzyme (CKMB, U/L), white blood cell count (WBC, ×10^9^/L), partial pressure of oxygen (PaO₂, mmHg), partial pressure of carbon dioxide (PaCO₂, mmHg), total bilirubin (TBIL, μmol/L), alanine aminotransferase (ALT, U/L), aspartate aminotransferase (AST, U/L), lactate dehydrogenase (LDH, U/L), blood urea nitrogen (BUN, mmol/L), serum creatinine (SCr, μmol/L), sodium (Na, mmol/L), lactate-to-alanine ratio (LAR), triglycerides (TG, mmol/L), total cholesterol (TC, mmol/L), and glucose (GLU, mmol/L). All vital signs and laboratory variables were defined using the earliest available measurements recorded after hospital arrival. These measurements were selected to represent the patient's initial physiological status during early trauma care. However, because some patients may have received prehospital or emergency interventions before these measurements were obtained, including supplemental oxygen, fluid resuscitation, vasoactive therapy, or mechanical ventilation, the potential influence of early treatment on these variables could not be completely excluded.

For mechanically ventilated patients, respiratory parameters and arterial blood gas values were recorded according to the clinical measurements available at the time of assessment. The study was designed to identify early prognostic markers in real-world trauma care rather than treatment-naïve physiological parameters.

### 2.4 Statistical analysis

#### 2.4.1 Descriptive analysis

In the descriptive analysis, continuous variables were expressed as medians with interquartile ranges (IQR) due to their skewed distribution. Categorical variables were presented as frequencies and percentages. To compare differences in variable distributions between the treatment success and treatment failure groups, the Mann–Whitney *U* test was used for continuous measures, while the chi-square test was applied for categorical variables.

#### 2.4.2 Predictive model development

The full dataset was randomly divided into a training set (223 patients, 70%) for model construction and a test set (95 patients, 30%) for hold-out test validation using outcome stratification. To address multicollinearity and identify features meaningfully associated with treatment failure, least absolute shrinkage and selection operator (LASSO) regression was performed on the training set (10). The optimal regularization parameter *λ* was selected through 10-fold cross-validation based on minimum binomial deviance. Features retained after LASSO filtering were included in subsequent predictive modeling.

For prediction, 5 ML algorithms were developed concurrently: Decision Tree (DT), Random Forest (RF), Support Vector Machine (SVM), k-Nearest Neighbors (KNN), and Extreme Gradient Boosting (XGBoost). Hyperparameter optimization for each model was conducted via a systematic grid search combined with 5-fold cross-validation (11). Once tuned, each model was retrained on the complete training set and evaluated rigorously on the test set. Performance was assessed using a suite of metrics, including the area under the receiver operating characteristic curve (AUC) with its 95% confidence interval (CI), accuracy, sensitivity, specificity, positive predictive value (PPV), negative predictive value (NPV), Kappa value, and the F1-score, with AUC serving as the primary indicator. We also built a logistic model on the same process as ML model. Additionally, decision curve analysis (DCA) was employed to quantify the clinical net benefit of each model across a range of probability thresholds, thereby evaluating practical utility. In addition, calibration curve analysis was employed to assess the agreement between a model's predicted probabilities and the truth outcomes. The model exhibiting the highest discriminative ability was selected for further investigation.

To improve model interpretability, SHapley Additive exPlanations (SHAP)—a method grounded in cooperative game theory—was utilized. This approach provides both a global ranking of feature importance and detailed insights into the direction and magnitude of each predictor's contribution to the risk of treatment failure (12).

All statistical and ML procedures were performed using R software (version 4.5.0). A two-tailed *P* value <0.05 was considered statistically significant.

## 3 Results

### 3.1 Baseline characteristics of study participants

Table 1 presents the baseline characteristics of 318 patients stratified by treatment outcome. Significant differences were observed between the treatment success and treatment failure groups across multiple variables. The variables showing statistically significant differences included MODS from disease status; disease severity scores (APACHE II, GCS, ISS); vital signs (P, R, T); and the majority of laboratory parameters: pH, K, LAC, RBC, HB, HCT, PLT, ALB, cTnI, MYO, TT, PT, APTT, Fib, DD, CK, CKMB, LAR, TG, TC, GLU. The remaining demographic characteristics, lifestyle factors, and some laboratory parameters showed no statistically significant differences between the two groups (all *P* > 0.05).

**Table 1:** Baseline characteristics of participants grouped by treatment.

Variables	Overall	Treatment Successful group	Treatment failure group	*P*
n	318	243	75	
Demographic characteristics				
Gender, *n* (%)				0.456
Male	225 (70.8)	175 (72.0)	50 (66.7)	
Female	93 (29.2)	68 (28.0)	25 (33.3)	
Age, [median (IQR), years]	50.50 (40.00, 62.00)	50.00 (40.00, 60.50)	52.00 (40.00, 65.50)	0.247
Lifestyle factors, *n* (%)				
Smoking status				0.114
Yes	65 (20.4)	55 (22.6)	10 (13.3)	
No	253 (79.6)	188 (77.4)	65 (86.7)	
Drinking status				0.209
Yes	46 (14.5)	39 (16.0)	7 (9.3)	
No	272 (85.5)	204 (84.0)	68 (90.7)	
Disease status, *n* (%)				
MODS				<0.001
Yes	82 (25.8)	23 (9.5)	59 (78.7)	
No	236 (74.2)	220 (90.5)	16 (21.3)	
Shock				0.772
Yes	189 (59.4)	146 (60.1)	43 (57.3)	
No	129 (40.6)	97 (39.9)	32 (42.7)	
Disease severity scores, median (IQR)				
APACHE score (scores)	12 (8, 19)	9 (6.5, 14)	21 (18, 24.5)	<0.001
GCS (scores)	10 (7, 15)	15 (10, 15)	4 (3, 5)	<0.001
ISS (scores)	29 (24, 36)	27 (22, 33)	40 (33, 45)	<0.001
Vital signs, median (IQR)				
P (beats per min)	92.5 (78.25, 114)	92 (78, 110)	104 (80, 122)	0.023
R (breaths per min)	20 (18, 23)	20 (18, 23)	18 (15, 24)	0.002
MAP (mmHg)	77.33 (64.5, 91.92)	77 (66, 90)	80.67 (62.66, 99.16)	0.421
T (℃)	36.1 (36, 36.5)	36.2 (36, 36.5)	36 (36, 36.5)	0.028
Laboratory parameters, median (IQR)				
pH	7.37 (7.32, 7.4)	7.37 (7.33, 7.4)	7.34 (7.24, 7.39)	0.002
K (mmol/L)	3.42 (3.1, 3.81)	3.46 (3.17, 3.84)	3.32 (2.8, 3.64)	0.004
LAC (mmol/L)	3.3 (2, 5.38)	2.9 (1.8, 4.5)	5.9 (3.55, 8.85)	<0.001
RBC (×10^12^/L)	3.2 (2.69, 3.78)	3.31 (2.76, 3.8)	2.91 (2.46, 3.42)	0.002
HB (g/L)	97 (81, 116)	100 (83, 118.5)	90 (76.5, 103.5)	0.002
HCT (%)	28.6 (23.63, 33.9)	29.2 (24.95, 34.65)	26.8 (22.15, 30.5)	0.001
PLT (×10^9^/L)	117 (78, 165.5)	131 (91, 177)	73 (51, 107)	<0.001
ALB (g/L)	31.15 (27.15, 35.48)	32.4 (28.2, 35.85)	28.9 (24.95, 33.5)	<0.001
cTnI (*μ*g/L)	0.06 (0.01, 0.35)	0.03 (0.01, 0.22)	0.34 (0.08, 0.87)	<0.001
MYO (μg/L)	933.15 (344.93, 1,200)	1,000 (375.15, 1,200)	479.8 (261.4, 1,083)	0.008
TT (s)	18.5 (16.7, 21)	18.2 (16.4, 20.15)	20.2 (17.6, 24.2)	<0.001
PT (s)	12.8 (11.7, 14.1)	12.5 (11.6, 13.7)	13.1 (12.25, 15.8)	<0.001
APTT (s)	27.45 (23.7, 33.88)	26.4 (23.3, 30.95)	34.6 (26.35, 44.15)	<0.001
Fib (g/L)	1.42 (1.14, 1.91)	1.49 (1.2, 2)	1.14 (0.84, 1.54)	<0.001
DD (mg/L FEU)	28.09 (9.79, 58.82)	21.2 (9.23, 48.42)	40 (14.33, 112.22)	<0.001
CK (U/L)	564 (302, 1,012)	623 (367.5, 1,112.5)	357 (251, 630)	<0.001
CKMB (U/L)	53 (34, 107)	47 (29, 85.5)	108 (52.5, 169.5)	<0.001
WBC (×10^9^/L)	12.71 (10.09, 17.34)	12.7 (10.27, 16.53)	13.54 (8.84, 18.9)	0.508
PaO_2_ (mmHg)	106 (74.55, 167.5)	106 (74.7, 157)	138 (75.1, 194)	0.067
PaCO_2_ (mmHg)	38.6 (34.5, 41.98)	38.6 (35.05, 41.1)	37 (31.05, 43.95)	0.290
TBIL (μmol/L)	13.9 (9.9, 18.1)	14.05 (10.35, 18.1)	12.2 (8.8, 18.1)	0.126
ALT (U/L)	41.75 (25, 75.75)	41.5 (25, 69.5)	43 (25.5, 120)	0.357
AST (U/L)	67.5 (40, 135.5)	68 (39, 120)	64 (41, 168)	0.526
LDH (U/L)	390.75 (292, 564.25)	390.5 (290, 563.5)	393 (308.5, 562)	0.558
BUN (mmol/L)	5.68 (4.69, 6.79)	5.71 (4.82, 6.97)	5.51 (4.6, 6.66)	0.252
SCr (μmol/L)	68 (57, 88.75)	67 (55, 87.5)	72 (59.5, 92.5)	0.210
Na (mmol/L)	141 (138.25, 143)	141 (139, 143)	140 (137, 144)	0.458
LAR	0.42 (0.32, 0.57)	0.4 (0.31, 0.52)	0.48 (0.33, 0.64)	0.025
TG (mmol/L)	0.76 (0.44, 1.15)	0.85 (0.46, 1.26)	0.6 (0.37, 0.81)	<0.001
TC (mmol/L)	2.98 (2.31, 3.78)	3.12 (2.41, 3.82)	2.6 (2.09, 3.46)	0.004
GLU (mmol/L)	7.81 (6.66, 9.88)	7.56 (6.53, 9.37)	9.14 (7.35, 12.02)	<0.001

### 3.2 Feature screening

**Figure 1:**
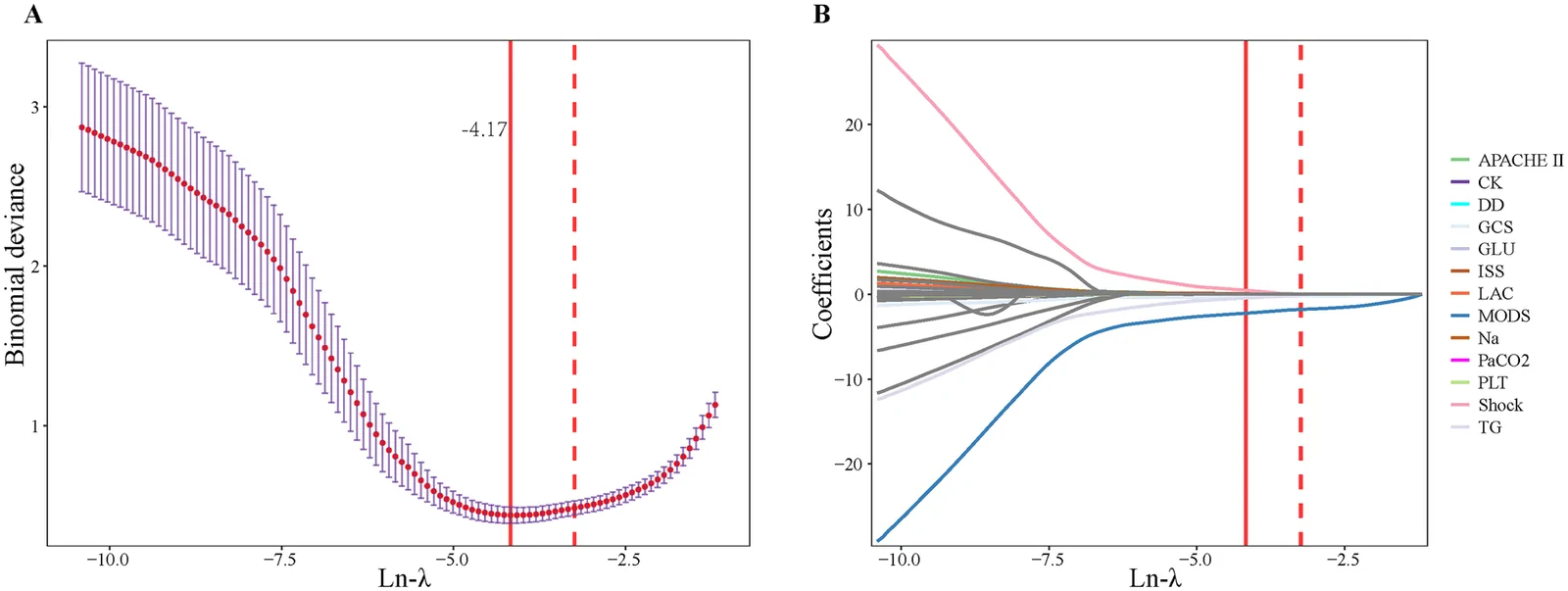
Variables selected based on LASSO regression in the training set. **(A)** The variation of binomial deviance with the Ln-*λ*. **(B)** The screening path of the LASSO regression for variables that are highly relevant to treatment failure.

Feature selection was performed using LASSO regression analysis (Figure 1). Panel A demonstrates the determination of the optimal natural logarithmic transformation (Ln-*λ*) value through 10-fold cross-validation, with the selection criterion being the minimization of binomial deviance. Panel B displays the LASSO coefficient path plot, illustrating the shrinkage of variable coefficients toward zero as the regularization parameter Ln-*λ* increases. The vertical dashed lines correspond to the Ln-*λ* values of −4.74 under the “minimum error”. Following the “minimum error” rule, a subset of variables with non-zero coefficients was selected from the initial feature, including APACHE II, CK, DD, GCS, GLU, ISS, LAC, MODS, Na, PaCO2, PLT, Shock and TG. These features were included in subsequent predictive modeling.

### 3.3 The application of ML models

**Figure 2:**
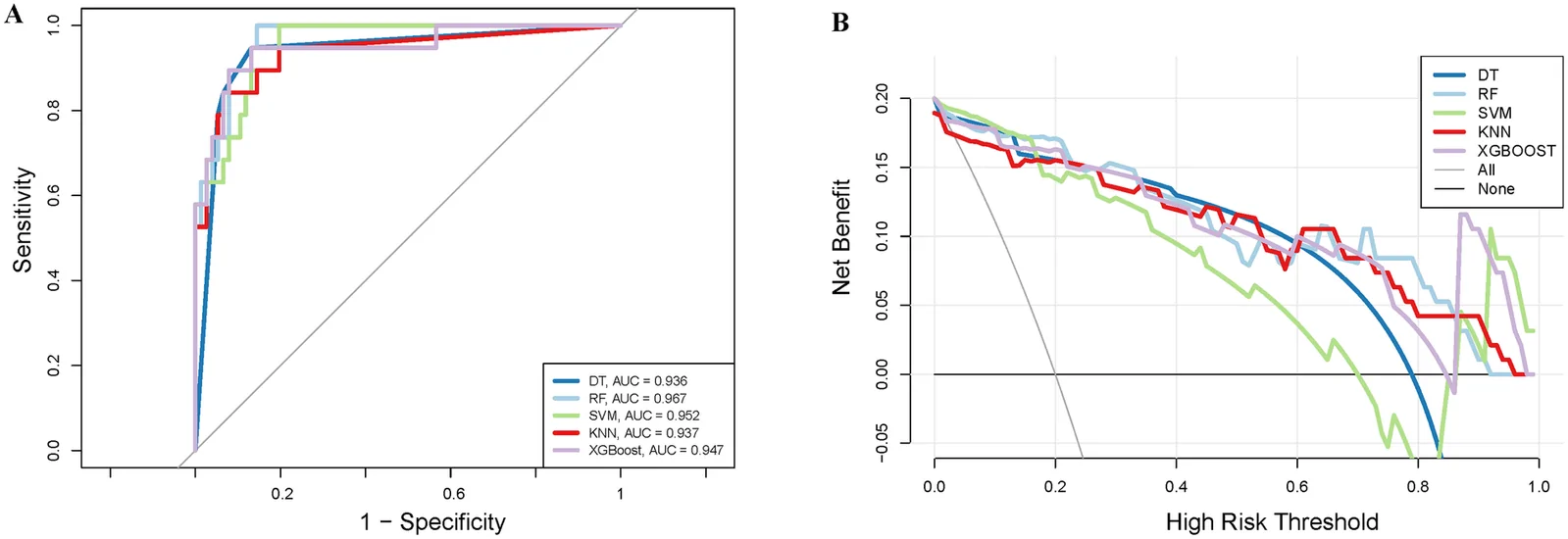
The predictive performance of 5 models on the test set using ROC curves and decision curve. **(A)** ROC curves. **(B)** Decision curves.

We constructed prediction models using 5 ML algorithms with their performance metrics detailed in Table 2. Best hyperparameter configurations for the 5 ML models are displayed in Supplementary Table S1. All models demonstrated excellent discriminative ability, with AUC ranging from 0.936 to 0.967. Among them, the RF achieved the highest AUC among all evaluated models, and was therefore selected as the primary model based on our pre-specified primary evaluation metric, achieving an AUC of 0.967 (95% CI: 0.936–0.997), an accuracy of 0.928, a sensitivity of 1.000, a specificity of 0.855, along with good clinical agreement (Kappa value = 0.703) and predictive balance (F1-score = 0.776). CIs of other performance metrics of 5 ML models are displayed in Supplementary Table S2. We comprehensively consider AUC and sensitivity, and ultimately determine RF as the optimal model. The receiver operating characteristic (ROC) curves for all five models, along with their decision curves, are illustrated in Figure 2. Based on decision curve analysis, each of the developed models offered a greater net clinical benefit compared to the two extreme scenarios—treating all patients or treating none—across a wide spectrum of clinically meaningful threshold probabilities. The performance of logistic regression is not as good as that of RF model (Supplementary Table S2). In the end, the RF model was identified as the optimal predictive model and was therefore retained for further interpretation and downstream analysis. Supplementary Figure S1 presents the calibration curve for the RF model, with predicted probabilities binned into 10 intervals. The calibration curve consistently fell below the ideal diagonal, reflecting a tendency to overestimate observed risks. The Brier score (0.084) indicated high predictive accuracy.

**Table 2:** The performance of 5 models in the test set.

Model	DT	RF	SVM	KNN	XGBoost
AUC (95% CI)	0.936 (0.874, 0.999)	0.967 (0.936, 0.997)	0.952 (0.911, 0.992)	0.937 (0.869, 1.000)	0.947 (0.886, 1.000)
Accuracy	0.908	0.928	0.901	0.888	0.908
Sensitivity	0.947	1.000	1.000	0.842	0.895
Specificity	0.868	0.855	0.803	0.934	0.921
PPV	0.643	0.633	0.559	0.762	0.739
NPV	0.985	1.000	1.000	0.960	0.972
Kappa value	0.693	0.703	0.619	0.747	0.756
F1 score	0.766	0.776	0.717	0.800	0.810

### 3.4 SHAP analysis

**Figure 3:**
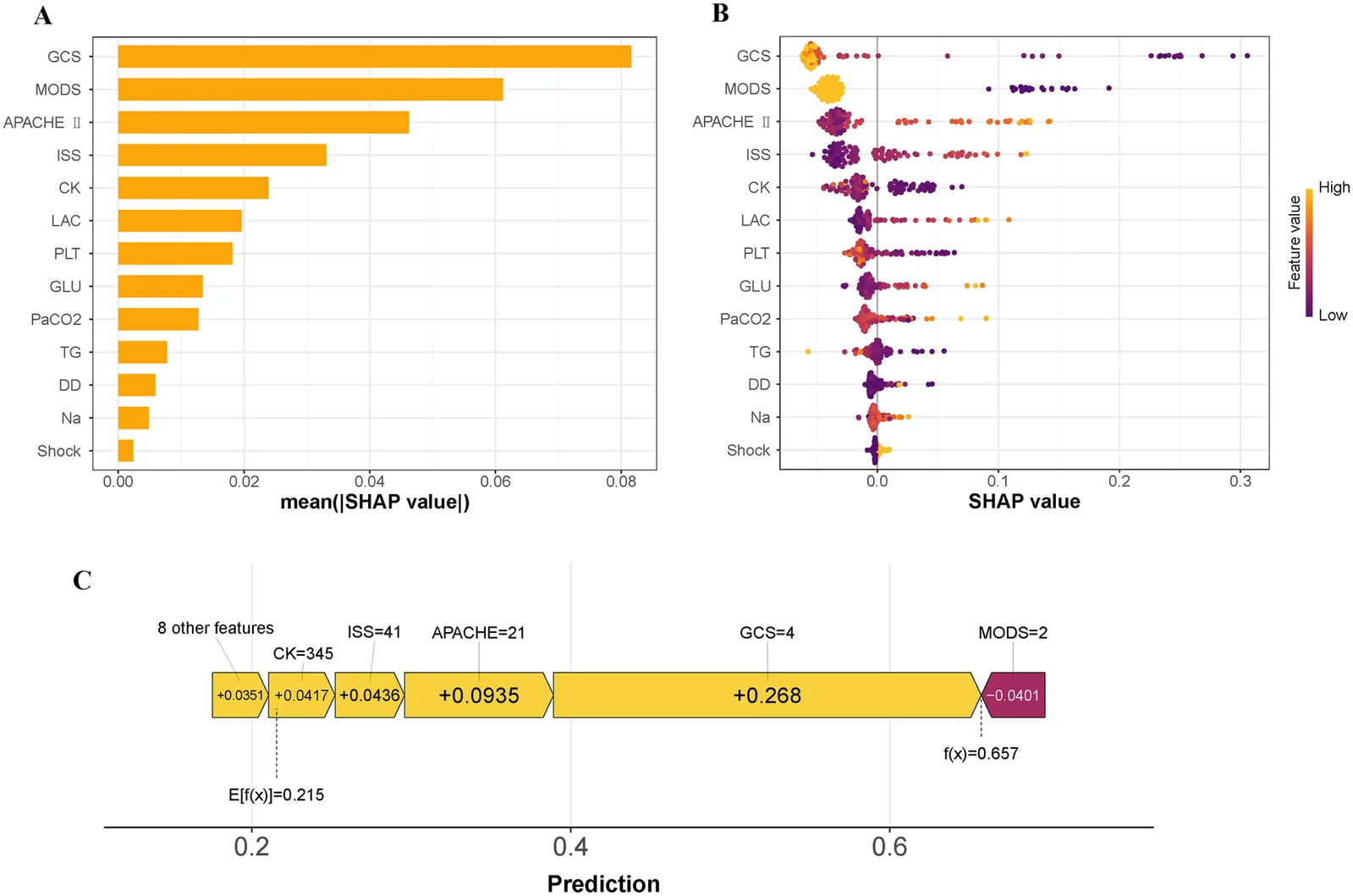
SHAP analysis based on RF model. **(A)** Feature importance plot. **(B)** Beeswarm plot. **(C)** Force plot for the prediction of random individual.

Figure 3 presents a comprehensive SHAP analysis of the clinical outcome prediction model. Panel A displays the global feature importance based on the mean absolute SHAP values. Among the evaluated clinical features, GCS score exhibited the highest mean SHAP value, indicating its greatest average contribution to model predictions, followed by MODS, APACHE II score, and ISS. Among the laboratory markers, CK demonstrated the largest mean SHAP value. Panel B illustrates the beeswarm plot, depicting the distribution of SHAP values for each feature. The color gradient from low (purple) to high (yellow) represents the actual feature value, illustrating how the magnitude and direction of feature influence depend on its numerical value. Higher APACHE II scores and ISS showed a clear positive correlation with treatment failure. Conversely, higher GCS score, without MODS, and higher CK level were associated with a lower risk of treatment failure. Panel C provides a local explanation for an individual prediction instance (force plot), demonstrating how each feature contributes to shifting the baseline prediction *E*[*f*(*x*)] = 0.215 to the final model output *f*(*x*) = 0.657. In this specific patient, a low GCS score (GCS score = 4) contributed the largest positive increment (+0.268), significantly elevating the predicted outcome. Other features such as APACHE II score = 21 (+0.0935), ISS = 41 (+0.0436), MODS = No (+0.0351), and CK = 345 (+0.0417) also contributed positively, while some features exerted a minor negative influence (e.g., −0.0401 from other aggregated features).

**Figure 4:**
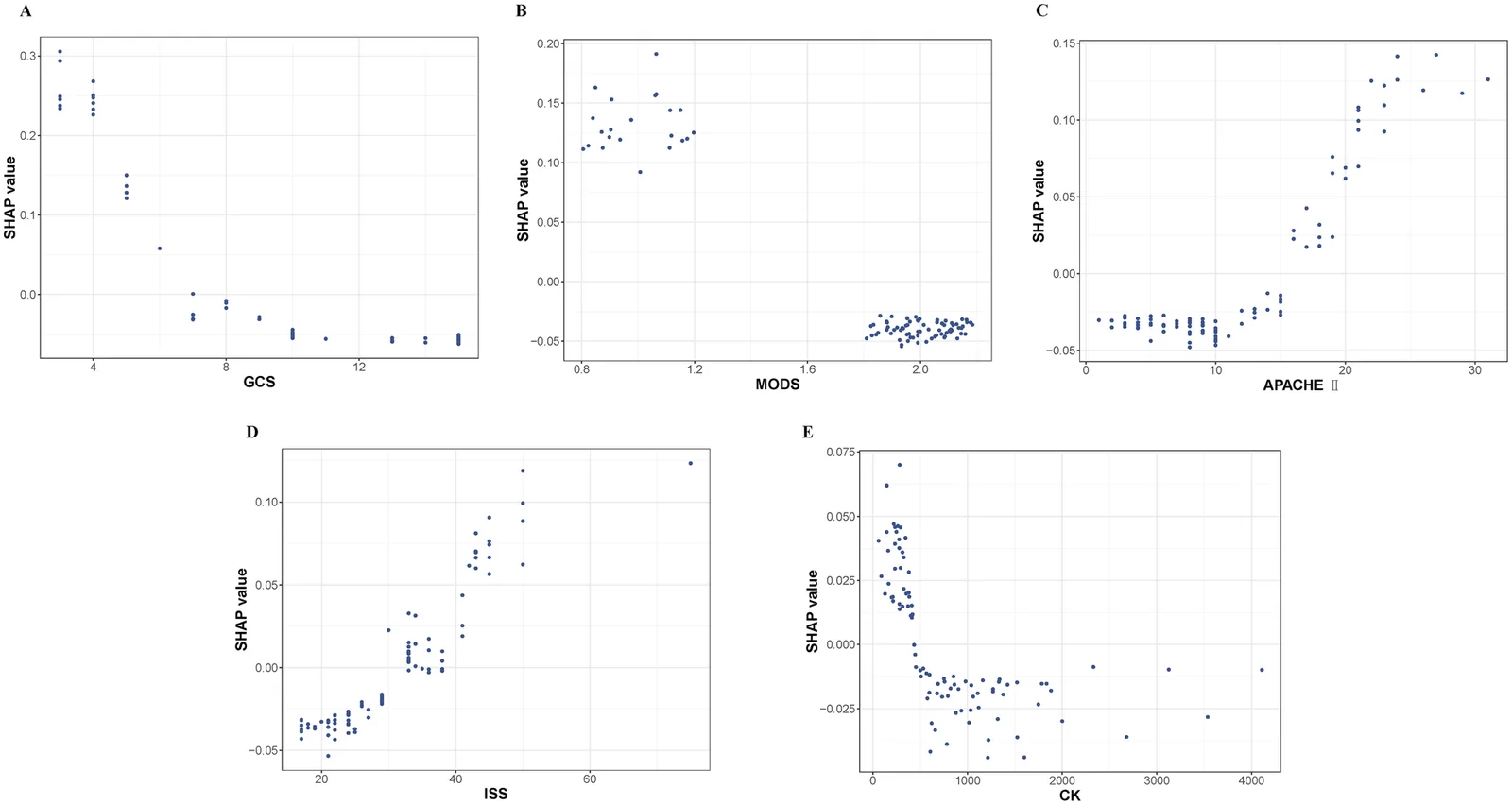
SHAP dependence plots about the correlation of 5 features and treatment failure. **(A)** GCS score. **(B)** MODS. **(C)** APACHE II score. **(D)** ISS. **(E)** CK.

Then, we used the SHAP dependence plots to explore the non-linear relationships between key continuous features and the predicted risk of treatment failure (Figure 4). Higher APACHE II score and ISS were still associated with an increased risk of treatment failure, while higher GCS score, absence of MODS, higher CK level were correlated with a reduced risk of treatment failure. These results indicated that there is no threshold effect of these important continuous features on risk treatment failure.

## 4 Discussion

In this study, we developed and validated a series of ML models to predict treatment failure in trauma patients using a comprehensive set of demograpHics, life style, clinical, and laboratory variables. Among the five algorithms evaluated, the RF model demonstrated the highest discriminative ability, achieving an AUC of 0.967. Notably, SHAP analysis provided both global and local interpretability, revealing that GCS score, MODS, APACHE II score, and ISS were the most influential features overall, while CK emerged as the most important laboratory predictor. This finding reinforces the central role of neurological status, systemic inflammation, and injury burden in determining clinical trajectories.

GCS score exhibited the predominant role in predicting treatment failure, and higher GCS score was consistently associated with a lower risk, which aligns with established neurotrauma pathopHysiology where impaired consciousness reflects severe brain injury, elevated intracranial pressure, or systemic hypoperfusion (13). A higher GCS score correlates with superior neurological function and an elevated level of consciousness, typically indicating a more favorable prognosis (1). An epidemiological study from India showed that GCS showed the best performance in predicting the outcome of patients with TBI compared with other scores (14). In a study investigating mortality predictors in pediatric trauma patients, the GCS score remained a prominent factor among all clinical characteristics. It emerged as an independent predictor of mortality, with only body temperature, age, and race demonstrating stronger associations with the outcome (15). When interpreting the importance of GCS features, it should be noted that the outcome of this study, “treatment failure”, includes the component of adverse outcomes of GOS, and GCS, as the most direct indicator of neurological function status in TBI patients, has an inherent clinical association with GOS. Therefore, the prominent position of GCS in SHAP importance ranking may be partly due to the overlap between GCS and outcome components, rather than fully representing its independent prognostic value. Our study shows that when predicting the prognosis of trauma patients, priority should be given to the GCS score. Nevertheless, the prognostic importance of GCS should be interpreted cautiously in intubated or sedated patients, in whom reliable assessment, particularly of the verbal component, may be challenging.

MODS is a critical and often sequential syndrome following major trauma, characterized by the failure of two or more organ systems, such as cardiovascular shock, respiratory failure, and neurological impairment (16). The presence of MODS was strongly associated with treatment failure, consistent with its definition as a syndrome of progressive pHysiological derangement across organ systems. APACHE II score is a commonly used indicator to quantitatively evaluate the severity of illness and predict the risk of death in critically ill patients (17). Previous studies have also shown that APACHE II is an effective predictor of 28 day in-hospital mortality and acute pancreatitis mortality in elderly patients with neurologic illness (18, 19). As an anatomical injury quantification tool, ISS demonstrated a consistent positive association with treatment failure, emphasizing that the overall anatomical injury burden remains an important determinant of prognosis. However, ISS considers only the highest AIS score within each anatomical body region and may therefore underestimate injury severity in patients with multiple severe injuries occurring within the same region. In contrast, the New Injury Severity Score (NISS) considers the three most severe injuries irrespective of anatomical region and may provide a more comprehensive representation of injury burden in such patients. As NISS was not available in the present retrospective dataset, future studies should evaluate whether incorporating NISS could further improve prognostic assessment and model performance.

Among all laboratory markers, CK contributed the most to the prediction. CK plays an important role in maintaining cellular energy homeostasis. CK mediates the rapid regeneration of adenosine tripHospHate (ATP) from adenosine dipHospHate (ADP) and pHospHocreatine, a reaction fundamental to sustaining the high-energy demands of muscular contraction, particularly within cardiac myocytes (20). Our SHAP analysis revealed a paradoxical negative correlation between initial CK levels and the risk of treatment failure. While extremely elevated CK is traditionally viewed as a marker of severe tissue damage or rhabdomyolysis, our finding aligns with the “energy buffer” hypothesis of the CK/pHospHocreatine (PCr) system. The CK system is fundamental for maintaining cellular energy homeostasis by facilitating the rapid regeneration of ATP from ADP and PCr, particularly in high-demand tissues like the myocardium and skeletal muscle during the hyperacute pHase of trauma. A relatively robust initial CK level may reflect a preserved metabolic “reserve” or the body's capacity to mount an energetic response to systemic stress. Conversely, depleted or abnormally low CK levels in critically ill trauma patients might indicate a state of metabolic exhaustion or mitochondrial dysfunction, leading to an inability to sustain vital organ function. This suggests that within the context of early risk stratification, the CK system's role in stabilizing mitochondrial function and reducing oxidative stress may be a key determinant of clinical resilience. By maintaining the energy flux between mitochondria and cytosol, CK system helps to stabilize mitochondrial function and reduce oxidative stress, and may indirectly exert anti apoptotic and anti-inflammatory effects by maintaining cellular energy homeostasis (21). Therefore, enhancing or protecting the function of CK system aims to improve the tolerance of tissues to ischemia and hypoxia from the level of energy metabolism, so as to maintain the function of key organs and ultimately improve the clinical prognosis (22). However, SHAP analysis only describes the statistical association patterns between variables and outcomes, and its essence is data-driven observational findings that cannot establish causal relationships or determine specific biological pathways. Therefore, the above mechanistic speculation about CK in this study should be considered as an exploratory hypothesis.

Our study has several strengths. First, we employed advanced ML technology to develop robust predictive models that effectively integrate a wide range of clinical and laboratory variables, enabling the identification of complex non-linear relationships that are often overlooked by traditional statistical methods. Second, the use of SHAP ensured model transparency, allowing clinicians to understand both “which” features matter and “how” they influence predictions.

Notably, several routinely collected physiological parameters, such as heart rate, respiratory rate, body temperature, and PaO₂, were initially considered but were not retained after feature selection, suggesting that these variables provided limited incremental predictive value within our modeling framework.

However, certain limitations should be acknowledged. The single-center, retrospective design may have introduced selection bias and limits the external validity of our findings. Additionally, the lack of external validation and comparison with conventional trauma models (e.g., TRISS, APACHE II, ISS) restricts the clinical applicability of our model. The modest cohort size (318 patients, with only 75 treatment-failure events) may have introduced instability and a risk of optimistic performance estimates, despite our use of LASSO regularization, repeated cross-validation, and bootstrap resampling to mitigate overfitting. The composite outcome “treatment failure” comprises multiple components with distinct pathophysiological mechanisms and clinical determinants—for instance, in-hospital mortality is often associated with irreversible injury or refractory shock, whereas unfavorable GOS primarily reflects neurological recovery. While aggregating these components was necessary to ensure an adequate number of events for model development, this may mask differential predictive performance across specific outcomes. Furthermore, the calculation of APACHE II and MODS may have introduced temporal leakage if these scores incorporated data not available at the intended prediction time point. Another is that several physiological and laboratory variables included in the candidate feature set may have been influenced by early trauma management. Interventions such as supplemental oxygen administration, mechanical ventilation, fluid resuscitation, and vasoactive therapy may alter parameters including arterial blood gases, lactate levels, and hemodynamic measurements. Although we used the earliest available measurements after hospital arrival, the retrospective design prevented us from fully determining the exact temporal relationship between each intervention and measurement. Therefore, the identified predictors should be interpreted as early prognostic markers rather than causal determinants of treatment failure. There is also another limitation concerns the assessment of GCS in patients who underwent endotracheal intubation or received sedative or neuromuscular blocking agents. Although we used the earliest reliable GCS documented before sedation or intubation whenever available, GCS assessment may still be less reliable in patients who were already intubated before hospital arrival, particularly because the verbal component cannot be directly assessed after intubation. The FOUR (Full Outline of UnResponsiveness) score, which does not rely on verbal responses and additionally evaluates brainstem reflexes and respiratory patterns, may provide a more appropriate neurological assessment in such patients. However, FOUR scores were not routinely collected during the study period and could not be reliably reconstructed retrospectively. Future prospective studies should consider incorporating the FOUR score, particularly for intubated patients with severe trauma. Potential selection bias may also have arisen from excluding patients who died before completion of clinical examinations, those with incomplete laboratory data, and those with major comorbidities. These exclusions, necessitated by the data requirements of the modeling approach, may limit the generalizability of our findings and warrant cautious interpretation. Furthermore, while SHAP enhances interpretability, the “black-box” nature of ML models remains a concern in high-stakes clinical environments. Future work should focus on real-time validation and integration of such models into clinical workflows, possibly as decision-support tools.

## 5 Conclusion

In conclusion, the Random Forest model demonstrated strong internal discriminative performance for predicting treatment failure in this single-center trauma cohort. SHAP-based interpretation identified GCS, MODS, APACHE II, ISS, and CK as the important predictors contributing to model predictions. However, external validation in multi-center prospective cohorts, calibration assessment, and ideally prospective testing are required before any clinical application.

## Data Availability

The datasets presented in this study can be found in online repositories. The names of the repository/repositories and accession number(s) can be found in the article/Supplementary Material.
