# Supplementary material for: Development and validation of an interpretable machine-learning model for predicting treatment failure in severe trauma patients
Source: Front Med (Lausanne). 2026 Sep 7;13:1919742. doi: 10.3389/fmed.2026.1919742 (PMC13590346; doi:10.3389/fmed.2026.1919742)
Supplement: Supplementary file 1 [file Supplementaryfile1.docx]

**Supplement Table 1.** Best hyperparameter configurations for the 5 ML models.

| Model | Parameter | Value |
| --- | --- | --- |
| Decision Tree (DT) | cp (complexity parameter) | 0 |
|  | minsplit (minimum node size before splitting) | 10 |
|  | maxdepth (maximum tree depth) | 10 |
| Random Forest (RF) | mtry (number of variables sampled per split) | 2 |
|  | num.trees (number of trees) | 1000 |
|  | min.node.size (minimum node size) | 4 |
| Support Vector Machine (SVM) | kernel | radial |
|  | cost (penalty parameter) | 0.001 |
|  | gamma (kernel coefficient) | 0.01 |
| K-Nearest Neighbors (KNN) | k (number of neighbors) | 19 |
| XGBoost | nrounds (number of boosting rounds) | 500 |
|  | max_depth (maximum tree depth) | 4 |
|  | eta (learning rate) | 0.25 |
|  | gamma (minimum loss reduction for split) | 0.45 |
|  | colsample_bytree (fraction of features per tree) | 0.05 |
|  | min_child_weight (minimum sum of instance weights in child) | 4 |

**Supplement Table 2.** CIs of other performance metrics of 5 ML models.

| Model | Accuracy | Sensitivity | Specificity | PPV | NPV | Kappa | F1 Score |
| --- | --- | --- | --- | --- | --- | --- | --- |
| DT | 0.908 (0.851, 0.952) | 0.947 (0.838, 1.000) | 0.868 (0.783, 0.939) | 0.643 (0.488, 0.789) | 0.985 (0.938, 1.000) | 0.693 (0.541, 0.818) | 0.766 (0.648, 0.886) |
| RF | 0.928 (0.876, 0.969) | 1.000 (1.000, 1.000) | 0.855 (0.762, 0.932) | 0.633 (0.492, 0.792) | 1.000 (1.000, 1.000) | 0.703 (0.535, 0.832) | 0.776 (0.649, 0.882) |
| SVM | 0.901 (0.835, 0.953) | 1.000 (1.000, 1.000) | 0.803 (0.702, 0.902) | 0.559 (0.418, 0.724) | 1.000 (1.000, 1.000) | 0.619 (0.462, 0.771) | 0.717 (0.589, 0.844) |
| KNN | 0.888 (0.824, 0.942) | 0.842 (0.681, 0.952) | 0.934 (0.862, 0.980) | 0.762 (0.591, 0.905) | 0.960 (0.882, 1.000) | 0.747 (0.605, 0.863) | 0.800 (0.681, 0.911) |
| XGBoost | 0.908 (0.849, 0.955) | 0.895 (0.782, 0.962) | 0.921 (0.847, 0.976) | 0.739 (0.592, 0.881) | 0.972 (0.918, 1.000) | 0.756 (0.608, 0.873) | 0.810 (0.702, 0.912) |

**Supplement Table 3.** The performance of logistic regression in the test set.

| AUC (95%CI) | Accuracy | Sensitivity | Specificity | PPV | NPV | Kappa | F1 score |
| --- | --- | --- | --- | --- | --- | --- | --- |
| 0.892 (0.873, 0.911) | 0.854 (0.782, 0.926) | 0.930 (0.876, 0.984) | 0.541 (0.443, 0.639) | 0.985 (0.961, 1.000) | 0.715 (0.649, 0.781) | 0.662 (0.546, 0.778) |  |

**Supplement Figure 1.** Calibration curve of RF model.
